# Using diffusion anisotropy to characterize neuronal morphology in gray matter: the orientation distribution of axons and dendrites in the NeuroMorpho.org database

**DOI:** 10.3389/fnint.2013.00031

**Published:** 2013-05-14

**Authors:** Mikkel B. Hansen, Sune N. Jespersen, Lindsey A. Leigland, Christopher D. Kroenke

**Affiliations:** ^1^Center for Functionally Integrative Neuroscience and MINDLab, NeuroCampus Aarhus, Aarhus UniversityAarhus, Denmark; ^2^Department of Physics and Astronomy, Aarhus UniversityAarhus, Denmark; ^3^Department of Behavioral Neuroscience, Advanced Imaging Research Center, Oregon Health and Science UniversityPortland, OR, USA; ^4^Division of Neuroscience, Oregon National Primate Research Center, Oregon Health and Science UniversityPortland, OR, USA

**Keywords:** neuron morphology, MRI, diffusion, simulation, kurtosis, cytoarchitecture, cerebral cortex

## Abstract

Accurate mathematical modeling is integral to the ability to interpret diffusion magnetic resonance (MR) imaging data in terms of cellular structure in brain gray matter (GM). In previous work, we derived expressions to facilitate the determination of the orientation distribution of axonal and dendritic processes from diffusion MR data. Here we utilize neuron reconstructions available in the NeuroMorpho database (www.neuromorpho.org) to assess the validity of the model we proposed by comparing morphological properties of the neurons to predictions based on diffusion MR simulations using the reconstructed neuron models. Initially, the method for directly determining neurite orientation distributions is shown to not depend on the line length used to quantify cylindrical elements. Further variability in neuron morphology is characterized relative to neuron type, species, and laboratory of origin. Subsequently, diffusion MR signals are simulated based on human neocortical neuron reconstructions. This reveals a bias in which diffusion MR data predict neuron orientation distributions to have artificially low anisotropy. This bias is shown to arise from shortcomings (already at relatively low diffusion weighting) in the Gaussian approximation of diffusion, in the presence of restrictive barriers, and data analysis methods involving higher moments of the cumulant expansion are shown to be capable of reducing the magnitude of the observed bias.

## Introduction

Quantitative characterization of the dependence of the diffusion-attenuated magnetic resonance imaging (MRI) signal intensity on diffusion sensitization strength and direction provides a non-invasive strategy to study cellular morphology of neurons and glia in brain tissue (Beaulieu, 2002; Le Bihan, 2003; Mori and Zhang, 2006). Diffusion tensor imaging (DTI) and variants of DTI for characterizing water diffusion in brain have been utilized in a wide range of studies directed at normal brain white matter (WM) anatomy, and studies of WM development and pathology (Le Bihan, 2003; Mori and Zhang, 2006; Wozniak et al., 2006). Recently, the potential of diffusion MRI for characterizing cell morphology within brain gray matter (GM) structures has also been the subject of increasing recognition. Two GM structures that have been shown to be particularly well suited for diffusion MRI-based study are the cerebral cortex (McKinstry et al., 2002; Maas et al., 2004; deIpolyi et al., 2005; Kroenke et al., 2007, 2009; Huang et al., 2008, 2009; Jespersen et al., 2010; Budde et al., 2011; Takahashi et al., 2011; Leuze et al., 2012) (and see Leigland and Kroenke, 2010 for review) and hippocampus (Zhang et al., 2002; Shepherd et al., 2006; Laitinen et al., 2010; Delgado y Palacios et al., 2011; Vestergaard-Poulsen et al., 2011). In both contexts, a prominent morphological feature is the apical dendrite of pyramidal neurons. Anisotropy in water diffusion in GM, first observed in (Thornton et al., 1997), tends to be oriented parallel to this dominant organization. Within the developing cerebral cortex, morphological differentiation is associated with a loss of water diffusion anisotropy (Leigland and Kroenke, 2010), and the trajectory of diffusion anisotropy changes in cortex has been demonstrated to be sufficiently sensitive to enable the detection of abnormal morphological development (Sizonenko et al., 2007; Bock et al., 2010). In the mature human cortex, high-resolution diffusion MRI has revealed depth dependent anisotropy patterns, where superficial layers preferentially show tangential diffusion, and deeper layers have both radial and tangential diffusion anisotropy depending on depth and cortical location (Leuze et al., 2012; McNab et al., 2013). Within the hippocampus, neuron morphological changes associated with the response to stress have been demonstrated to be detectable with diffusion MRI (Delgado y Palacios et al., 2011; Vestergaard-Poulsen et al., 2011).

In order to facilitate the interpretation of diffusion MRI data in terms of underlying anatomical properties of cells in GM, modeling plays an important role. A successful model of the diffusion weighted signal must be based on realistic assumptions, and facilitate tractable and physically transparent analyses. For complex tissue such as the brain, it is challenging to avoid introducing overly simplistic assumptions about tissue structures. Therefore, it is necessary to incorporate simplifications into a modeling strategy while retaining the features of most importance for the diffusion signal. While physical intuition can guide the development of the model, proper subsequent testing, and validation of the model is clearly crucial.

We have previously proposed a biophysical model that relates the observed MRI signal to microstructural parameters including neurite volume fraction, intrinsic diffusion anisotropy within cellular axon/dendrite processes as well as the organization of cellular processes (Kroenke et al., 2004; Jespersen et al., 2007, 2012). The fundamental assumption of the model is that diffusion can be described in terms of two non-exchanging components. One component is associated with diffusion in cylindrically symmetric structures, such as cell processes with exchange of water being sufficiently slow to be considered impermeable on the time scale of the diffusion experiment. Dendrites and axons, collectively termed neurites, were assumed to fulfill these assumptions. The second component of the diffusion signal accounts for diffusion everywhere else, in particular in cell bodies, extracellular space, and glia cells. Here diffusion is assumed to be hindered, and molecular displacement is approximated to be a Gaussian function of displacement distance. The latter component is characterized by an effective diffusion tensor. This model has been shown to fit diffusion-weighted MRI data well (Jespersen et al., 2007) and to compare to histology and stereology with good agreement (Jespersen et al., 2010). More recently, experimental validation was sought for the ability to characterize the neurite orientation distribution, a characteristic of cellular morphology, using diffusion MRI data (Jespersen et al., 2012). This was done by expressing the orientation distribution of axonal and dendritic processes as a scatter matrix (or orientation matrix), and defining fractional anisotropy (FA) in the scatter matrix by reference to its eigenvalues in a manner analogous to DTI calculations. In the regime in which molecular displacement is Gaussian, *FA* in water diffusion is predicted to be linearly related to *FA* in the scatter matrix, and a linear relationship was observed between experimentally determined scatter matrices and diffusion tensors in post mortem brain tissue (Jespersen et al., 2012). However, the limitations of the Gaussian approximation in the context of comparing scatter matrices to diffusion MRI data have yet to be characterized.

The aim of the current work is to further develop the theory linking diffusion MRI data to neuron morphology by examining the Gaussian regime predictions in (Jespersen et al., 2012) for the intra-neuronal water component using numerical simulations. Specifically, the goodness-of-fit of the diffusion tensor model, which follows from the Gaussian phase approximation, is known to increase with decreasing *b*-value (Stepišnik, 1999; Sukstanskii and Yablonskiy, 2002; Zielinski and Sen, 2003; Kiselev, 2011). In order to characterize this phenomenon, we use the NeuroMorpho.org database (http://www.neuromorpho.org), which is a centralized repository for 3D recordings of neural morphologies (Ascoli, 2006; Ascoli et al., 2007), to obtain digital representations of real neurons, allowing us specifically to address the model simplifications concerning the geometric structure of neurons as collections of long cylinders. This approach has at least three advantages: (1) by focusing on only the intra-cellular compartment, we avoid possible confounding with other simplifications in the modeling, (2) the model component is tested under conditions representative of GM and (3) the ground truth is known.

## Methods

### Determination of the scatter matrices, T, for neocortical neurons in the NeuroMorpho database

We previously proposed a relationship between diffusion weighted MRI measurements and the distribution of cellular process orientations in brain tissue (Jespersen et al., 2012). Quantification of the orientation distribution of cellular processes is facilitated by the scatter matrix, T (Fisher and Embleton, 1987), which by the theory in Jespersen et al. (2012) is related to the diffusion tensor D. A complete description of diffusion in biological tissue is clearly of a non-gaussian nature (e.g., Mitra and Halperin, 1995; Stepišnik, 1999; Sukstanskii and Yablonskiy, 2002; Jespersen et al., 2007; Ozcan, 2010; Kiselev, 2011); nevertheless, the diffusion tensor remains a well-defined quantity which can be estimated from the cumulant expansion (Kiselev, 2011). Herein, scatter matrices are determined from the axonal and dendritic arbors of each neocortical neuron obtained from the NeuroMorpho.org database^1^, version 5.4 (Ascoli, 2006; Ascoli et al., 2007). Neuron reconstructions were downloaded from NeuroMorpho.org in SWC format (see Cannon et al., 1998; Ascoli, 2006; Ascoli et al., 2007, as well as the website, for a definition of this file structure), along with relevant reconstruction metadata, e.g., species, neuron type, laboratory of origin, etc. In order to retrieve the metadata for each neuron, a custom Internet information harvester written in python was created, and a local database connecting the neuron reconstruction data with the metadata was made. We have chosen to focus on a subset of the available 4639 neocortical neurons, yielding a total of 4558 neurons, distributed as: Human: *N* = 2147, monkey: *N* = 360, rat: *N* = 936, mouse: *N* = 1019, cat: *N* = 20, and elephant: *N* = 76.

**Figure 1:**
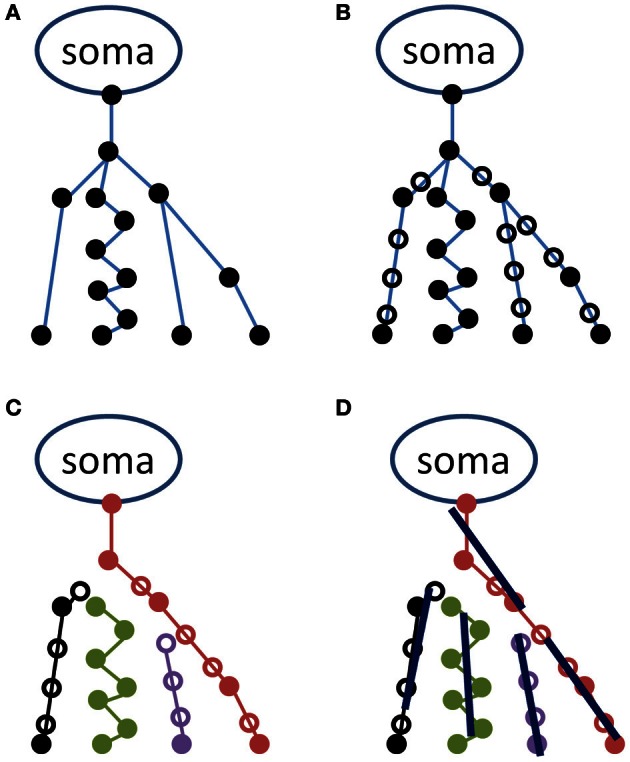
**Schematic representation of the procedure used in representing the neuronal tree by average lines.** A hypothetical neuron is illustrated in the upper left panel **(A)** in the original NeuroMorpho format as a series of coordinates (filled circles) connected as indicated by blue lines. In the upper right **(B)**, additional points have been inserted (open circles) for making the distance between two points less than or equal to a minimal distance (herein, 1 μm). In the lower left panel **(C)**, the neuron is represented as a set of unbranched paths, and in the lower right **(D)**, these are represented by average lines, each of length *l*.

The procedure for converting the raw data obtained in SWC fileformat to scatter matrices consists of four steps, and was implemented using Matlab (The Mathworks, Boston, MA). A schematic of the process for the initial steps is provided in Figure 1. In the first step, a data structure is created, in which each of its elements corresponds to a segment of an axon or dendrite, and the information contained in each data structure element includes, among other relevant characteristics, the beginning and ending coordinates of a line segment, and the identities of all “children” segments that emanate from the end coordinate. In Figure 1A, a hypothetical neuron represented by such a data structure is illustrated. Due to the close similarity between the data structure and the organization of the NeuroMorpho.org SWC file format, data structures that correspond to Figure 1A can be created by serially interpreting consecutive lines of a SWC text file. We note here that the conversion from raw data to the tree structure has been verified by computing the same measurements as is available in the metadata, e.g., the number of stems and branches, total length/surface/volume, maximum distances, and path lengths, etc.

As indicated in Figure 1A, neuronal process segments vary in length. This variation is potentially problematic in a later step of the analysis, in which all coordinates along a specified length (defined as the line length, *l*) are used to determine a straight line whose orientation will contribute toward the scatter matrix. Therefore, the second step of the analysis is to interpolate each segment in steps of distance less than *l*. Herein, neural processes are interpolated in 1 μm increments. Open symbols in Figure 1B indicate locations of interpolated coordinates.

The third step of the procedure is to convert the interpolated data structure to a list of unbranched paths (see Figure 1C). This is accomplished through a recursive process that begins by recording the coordinate of the “parent” node (the upper-most node in each of the Figure 1 panels), and descending through children nodes until a terminal node (i.e., a coordinate with no children segments) is reached. Each path in the list consists of a series of consecutive coordinates. The first path is generated by beginning at the parent node, determining if there are one or more children segments, and if there is only one, the coordinate corresponding to the child segment is added to the path. If there is more than one, the child segments are sorted in an arbitrary order, and the first child that has not already been incorporated to a path is added to the current path. Once a terminus is reached, a new path is initiated at a new parent node.

**Figure 2:**
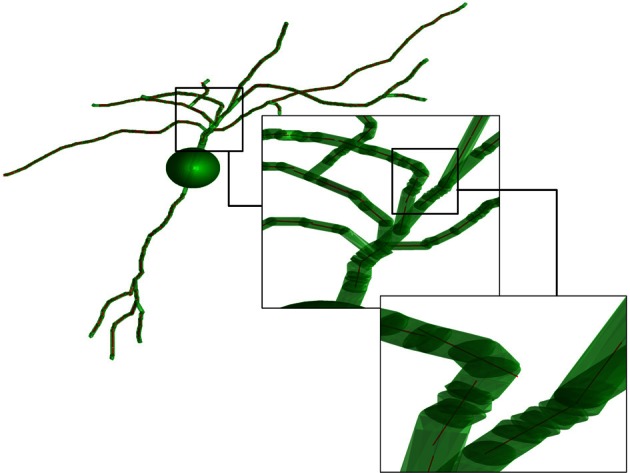
**Illustration of the representation of the neurons.** The transparent green cylinders represent the axons and dendrites, while the red lines represent the average line segments. A line segment length of 5 μm has been used. The image is based on a neocortex neuron from a 44 days old rat [entry P44_DEV203 of the NeuroMorpho.org database (Furtak et al., 2007)].

In the fourth step, the obtained set of paths is used to create a number of average line segments representing the neuronal tree. For each path, beginning with the first coordinate, consecutive coordinates are queried until the cumulative length of inter-coordinate line segments exceeds the line length *l*. Orthogonal distance regression, defined in Equation 18 of Jespersen et al. (2012), is then used to determine a single line that is closest to intersecting all points queried along the path. That process is repeated for all remaining segments of length *l* on the path, and subsequent paths from the neuron structure are similarly used to generate additional line segments. In general for the termini of each branch, there are a number of points that span a path length less than *l* that are not used to generate a line segment. Figure 2 illustrates the result of the Figure 1 procedure for a neuron reconstruction obtained from NeuroMorpho.org, in which a neuron (green surface) is approximated by a series of linear segments (red lines).

The scatter matrix is determined from the set of *N* line segments obtained from a neuron (*N* is the number of line segments). If the direction of each line segment *k* is expressed as a unit column vector, *u*_*k*_, the scatter matrix can be obtained from an *N* × 3 matrix S with row vectors $\sqrt{w_{k}}u_{k}^{T}$ in which *w*_*k*_ is a scalar weight computed as the volume fraction of a capped cylinder (volume π*lr*^2^_*k*_) corresponding to line segment *k*, i.e., *w*_k_ = *r*^2^_*k*_/∑_*i*_*r*^2^_*i*_, and where it is used that all line segments share a common length, *l*. The scatter matrix is then computed using the relationship T = S^T^S. Subsequent steps of the analysis are identical to analogous steps of the procedure for analyzing Golgi-stained neurons (Jespersen et al., 2012).

### Computing the diffusion MRI signal

The diffusion equation with appropriate boundary conditions has been solved for a small number of geometries as a means of modeling restrictive barriers to diffusion. One such geometry is the cylinder. Therefore, to take advantage of the known geometry of neurons in the NeuroMorpho database, explicit diffusion simulations were performed using boundary conditions appropriate for local cylindrical symmetry. For a Stejskal-Tanner diffusion measurement, given a diffusion-sensitization magnetic field gradient pulse $q=\gamma g\delta \overset{\text{def}}{=}q\hat{n}$; in direction $\hat{n}$, with γ the gyromagnetic ratio of the nuclei under consideration, **g** being the strength and direction of the magnetic gradient field, and δ being the duration of the pulse, the diffusion signal *S* may be computed by (Jespersen et al., 2007)

(1) $$S_{c}(q,\;\Delta )=\int_{S_{2}}d\hat{u}\;f(\hat{u})e^{-bD_{T}}e^{-{\left(\hat{u}\cdot \hat{n}\right)}^{2}\left(D_{L}-D_{T}\right)b}$$

In the above expression, *S*_2_ is an integration surface (the sphere), *û* is a unit direction vector for the local axis of symmetry for a given axonal or dendritic process, *f*(*û*) is a direction distribution function for the neural processes, *b* = (Δ−δ/3) *q*^2^, and Δ is the time between two gradient pulse onsets. In recognition of the local cylinder symmetry of neural axonal and dendritic processes, the intracellular diffusion coefficient is represented by a longitudinal part (parallel to the neural process) and a transverse part (perpendicular to the neural process) denoted *D*_*L*_ and *D*_*T*_, respectively. To simplify notation, an anisotropic diffusion coefficient is defined as *D*_*A*_ = *D*_*L*_ − *D*_*T*_. Note that Equation (1) is obtained from Jespersen et al. (2007) by setting the volume fraction of the neuronal compartment ν equal to one. For the simulations performed herein, the diffusion magnetic resonance (MR) signal may be obtained from a weighted sum over the *N* line segments [rather than from the integral expression in Equation (1)].

(2) $$S_{c}(q,\;\Delta )=\sum_{k=1}^{N_{1}}w_{k}e^{-bD_{T,\;k}}e^{-{\left({\hat{u}}_{k}\cdot \hat{n}\right)}^{2}\left(D_{L}-D_{T,\;k}\right)b}$$

in which *k* iterates over the N cylinders, $\hat{\;u_{k}}$ is the direction of cylinder *k*, and *w*_*k*_ is the weight factor, given by the volume fraction of cylinder *k*, as discussed previously. *D*_*T*, *k*_ is a transverse diffusion coefficient, which is estimated by considering the restricted 2D self-diffusion in a circle with radius given by cylinder *k*. The formula for computing *D*_*T*, *k*_ is derived based on the work of Stepišnik (1993), and is detailed in Appendix A. In this work we use a number of different gradient tables in the diffusion MRI signal generation, in order to illustrate different aspects of the underlying assumptions of the diffusion models to be described. Common to the gradient tables is that they consist of 63 directions for the non-zero *b*-values.

In order to estimate the diffusion tensor D, a diffusion model is fitted to the diffusion signal computed by Equation (2). In this paper we consider two models, (1) a diffusion tensor model and (2) a fourth order cumulant model (kurtosis model), see e.g., (Liu et al., 2004; Jensen et al., 2005; Lu et al., 2006; Jensen and Helpern, 2010; Kiselev, 2011). The models are given as

(3) $$S_{DTI}(q,\;\Delta )=S_{0}e^{-b_{ij}D_{ij}}$$

(4) $$S_{cum}(q,\;\Delta )=S_{0}e^{-b_{ij}D_{ij}}e^{-b_{ij}b_{kl}K_{ijkl}}$$

where summation over repeated indices is assumed, *b*_*ij*_ = (Δ − δ/3) *q*_*i*_*q*_*j*_, and where *D*_*ij*_ and *K*_*ijkl*_ are the *ij*'th and *ijkl*'th elements of the diffusion and kurtosis tensors, respectively. For convenience, we here absorb some front factors into the definition of the kurtosis tensor, as compared to Jensen et al. (2005). The models are fitted using the least squares curve fitting function available in MATLAB (2011). We note that the DTI model is expected to be valid only for low *b*-values, and hence the results presented in this work are based on a set of *b*-values ranging from 0 to 1 ms/μm^2^, unless stated otherwise. For the kurtosis model, we simulate the same experimental settings.

The translation of SWC files to scatter matrices, diffusion signals, and diffusion tensors has been implemented in MATLAB, where software has been written such that a set of neurons from the aforementioned database structure may be processed in a parallel framework.

In summary, we thus have access to the orientation distribution tensor T and the diffusion tensor D. These are readily diagonalizable, as they are symmetric 3 × 3 matrices and thus have real eigenvalues. From Equation (10) in Jespersen et al. (2012) with ν = 1, we note that the centralized eigenvalues of the two matrices are related through the anisotropic diffusion coefficient, as

(5) $$\left(\lambda_{i}-\bar{\lambda })=D_{A}(\tau_{i}-\bar{\tau }\right)$$

where λ_*i*_ is the *i*'th eigenvalue of the diffusion tensor, $\bar{\lambda }$ is the mean of the diffusion tensor eigenvalues, τ_*i*_ is the *i*'th eigenvalue of the orientation distribution matrix, and $\bar{\tau }$ is the mean of the eigenvalues of the orientation distribution matrix. Note that $\bar{\tau }$ always equals 1/3 by construction. From the eigenvalues, one may also compute the FA, which for the scatter matrix is given by

(6) $$FA_{T}=\sqrt{\frac{3}{2}\frac{{\left(\tau_{1}-\bar{\tau }\right)}^{2}+{\left(\tau_{2}-\bar{\tau }\right)}^{2}+{\left(\tau_{3}-\bar{\tau }\right)}^{2}}{\tau_{1}^{2}+\tau_{2}^{2}+\tau_{3}^{2}}}$$

in analogy to the diffusion tensor fractional anisotropy *FA*_*D*_ (Basser and Pierpaoli, 1996). With these definitions, it is a simple matter to relate the anisotropy from the diffusion tensor to the one for the orientation matrix (Jespersen et al., 2012), the result being

(7) $$FA_{D}\sqrt{\lambda_{1}^{2}+\lambda_{2}^{2}+\lambda_{3}^{2}}=D_{A}FA_{T}\sqrt{\tau_{1}^{2}+\tau_{2}^{2}+\tau_{3}^{2}}$$

As a final comment we note that the cell somas are treated as isotropic diffusion media (in Figure 2, the cell soma is represented by a sphere), which does not contribute to the separation of transverse and longitudinal diffusion. As a natural consequence, the cell somas are not included in the calculation of the diffusion signal.

To assess the influence of non-gaussian effects on the comparison between diffusion tensor and scatter matrix eigenvalues, an additional set of simulations were performed. In these, the MRI signal was calculated on the basis of a distribution of infinitely long and narrow cylinders with a diffusion coefficient D equal to 1 μm^2^/ms. We used the Gaussian approximation for diffusion in each single cylinder; thus, the signal contribution from a cylinder pointing along the direction *û* is $\exp(-bD{\left(\hat{n}\;\cdot \;\hat{u}\right)}^{2})$ when diffusion weighting *b* is applied along $\hat{n}$. The diffusion signal was then computed using 10,000 such cylinders with directions *û* randomly drawn from a Watson distribution (Fisher and Embleton, 1987; Jespersen et al., 2012)

(8) $$f(\hat{u})\propto \exp\left(\kappa {\left(\hat{u}\;\cdot \;\hat{z}\right)}^{2}\right)$$

with a given concentration parameter κ and principal orientation $\hat{z}$; and this process was repeated for 191 concentration parameters ranging from 1 to 20. The apparent diffusion coefficient, obtained by fitting a monoexponential decay in signal intensity with *b*-value, in a direction parallel to $\hat{z}$, is equal to the largest eigenvalue of the diffusion tensor, and the apparent diffusion coefficient perpendicular to the principal orientation is equal to the smallest two eigenvalues (for positive Watson concentration parameters as used here). Correspondingly, the largest eigenvalue τ_1_ of the scatter matrix is determined by numerically averaging ${\left(\hat{u}\;\cdot \;\hat{z}\right)}^{2}$ over the 10,000 cylinder directions, whereas the other two eigenvalues are determined using τ_2_ = τ_3_ = (1 − τ_1_)/2. The resulting characterization of diffusion and scatter matrices enables an additional and independent evaluation of Equations (5) and (7).

## Results

Prior to using neuron geometries available through the NeuroMorpho database for simulations and validation studies, the potential dependence of scatter matrix determinations on the line length chosen for approximating the reconstructions, as well as variability in neuron structure within the database, was characterized. Of the 4462 neocortical neurons in the NeuroMorpho database, 82 were excluded from the analyses presented here because the Figure 1 procedure yielded fewer than 100 line segments for these relatively small neurons, and statistical analysis based on this small number was considered unreliable. The following results are based on the remaining 4380 neurons (98% of the initial pool).

### Dependence of T and D on average line segment length

**Figure 3:**
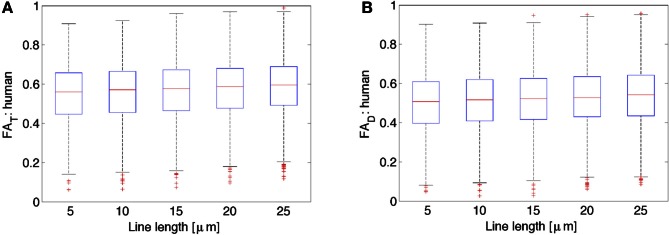
**Variation of *FA*_*T*_ or the orientation matrix fractional anisotropy **(A)** and *FA*_*D*_ or the diffusion tensor fractional anisotropy **(B)** as a function of the line length used to represent the neurons.** While there is a slight increase in both *FA*_*T*_ as well as *FA*_*D*_ with increases in line length, these data suggest that there is very little variation in both *FA*_*T*_ and *FA*_*D*_ due to differences in line length used to represent the neurons.

Figure 3 shows *FA* of the diffusion tensor and orientation distribution matrices for the human neurons (*N* = 2147) obtained from NeuroMorpho.org, as a function of the average length of the lines representing the neuronal tree. By inspection of Figure 3, it is clear that the *FA* is nearly constant with respect to the length of the lines used to represent the neurons. In general, both the *FA* obtained from the diffusion tensor and the one from the scatter matrix increase slightly as one increases the length of the path one averages over. Specifically, the increase is characterized by a linear slope of 0.0017 per μ m for *FA*_*D*_ from both the DTI and kurtosis models (the latter not shown), and 0.0018 per μ m for *FA*_*T*_. Although this dependence is extremely weak, it is significant due to the ability to characterize a large number of neurons (e.g., for *FA*_*T*_, *r* = 0.084, *p* < 0.0001). This weak dependence might be a result of the distribution of lines becoming less scattered, and thereby less isotropically distributed on a sphere. This potential trend is consistent with the expected result for the limiting case of approximating an arbitrary neuronal tree by a single line, in which the scatter matrix has two zero eigenvalues, yielding unit (i.e., maximal) FA. Similar results as the ones presented for the human neocortical neurons were also obtained for other species (data not shown). Given the weak dependence of *FA*_*T*_ and *FA*_*D*_ on line length, subsequent calculations presented here have utilized a line length of 10 μm, as was done in earlier studies (Jespersen et al., 2012).

### Dependence of T on species, neuron type, and laboratory of origin

**Figure 4:**
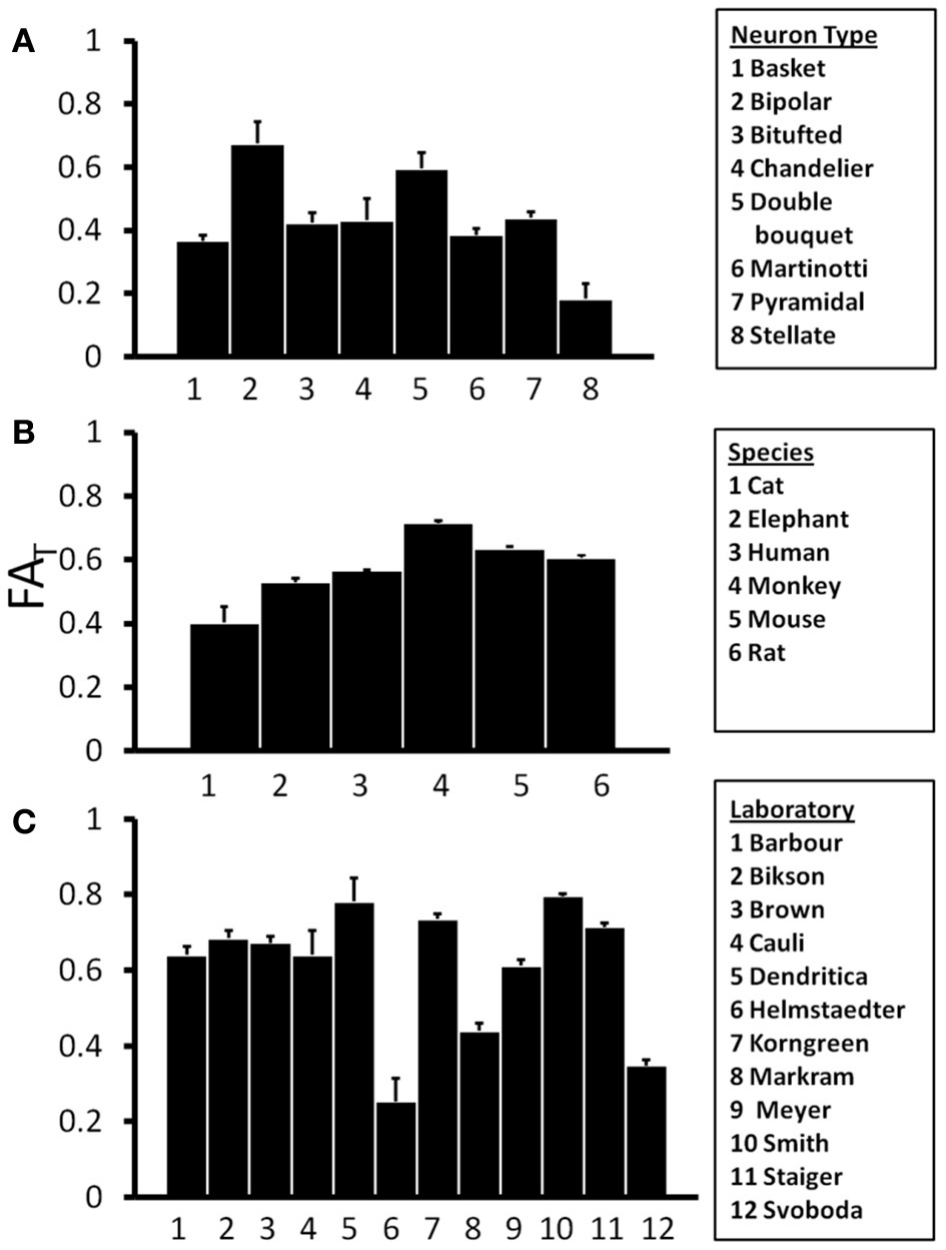
**One-Way Analysis of Variance (ANOVA) tests were performed to determine the effects of independent variables Neuron Type **(A)**, Species **(B)**, and Laboratory **(C)** on the orientation matrix fractional anisotropy, *FA*_*T*_.** ANOVA results revealed significant effects of all three independent variables on *FA*_*T*_. This suggests that variability in *FA*_*T*_ is potentially introduced by differences among neuron types, species, and laboratories.

If the shapes of neurons differ with respect to species or neuron type, such factors could influence water diffusion anisotropy measured in the cerebral cortex, and such a dependence would be of interest in the interpretation of diffusion MRI data. In Figure 4A, mean and standard error in *FA*_*T*_ for the various subtypes of rat neocortical neurons obtained from the research study that reported the largest number of neurons (“Markram”) are presented, and Analysis of variance (ANOVA), with a significance level of α = 0.05, reveals a significant effect of neuron type on *FA*_*T*_ [*F*_(7, 196)_ = 6.22, *p* < 0.0001]. This dependence on neuron type is also observed when neocortical neurons of all species, contributed by all laboratories, are pooled [*F*_(18, 4204)_ = 28.8, *p* < 0.0001]. The mean and standard error in *FA*_*T*_ for pyramidal neocortical neurons are shown for the six species in the NeuroMorpho database in Figure 4B. ANOVA reveals an additional statistically significant effect of species [*F*_(5, 3451)_ = 60.4, *p* < 0.0001] on *FA*_*T*_ values. The observed sensitivity of *FA*_*T*_ to the factors analyzed in Figures 4A,B provoked the question of whether systematic differences in *FA*_*T*_ exist between neurons reconstructed from different laboratories. In order to control for effects due to species and neuron type, mean, and standard error values for rat pyramidal neocortical neurons are shown in Figure 4C. Significant inter-laboratory variability is observed [ANOVA *F*_(11, 443)_ = 86.7, *p* < 0.0001], which indicates that inter-laboratory differences, perhaps resulting from different techniques utilized, appear to contribute to *FA*_*T*_ variability.

### Comparison to simulated diffusion MRI data

**Figure 5:**
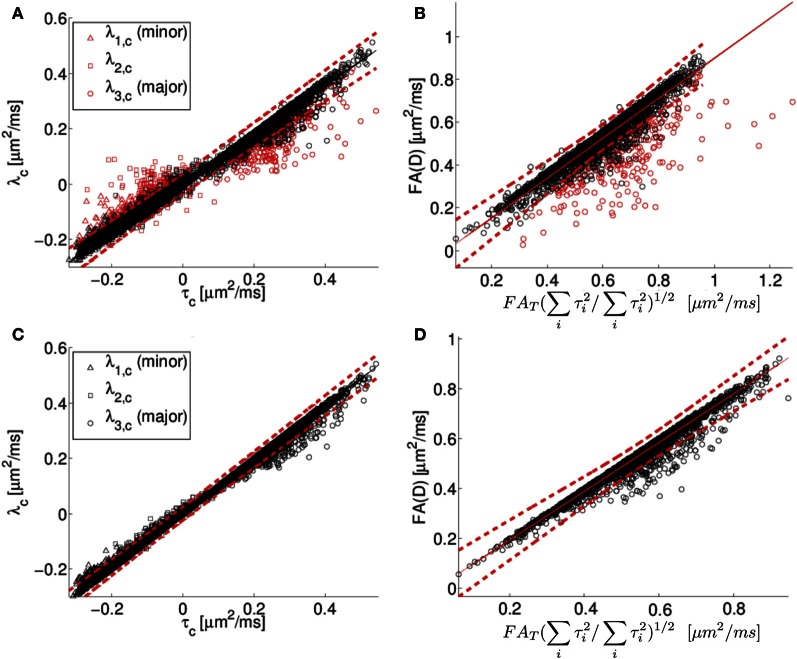
**In **(A)**, the centralized eigenvalues (λ_1_, λ_2_, λ_3_) of the diffusion tensor matrix (D) are plotted as a function of the corresponding eigenvalues of the orientation distribution matrix (T).** In **(B)**, the diffusion tensor fractional anisotropy (*FA*_*D*_) is plotted as a function of a scaled version of the orientation matrix fractional anisotropy (*FA*_*T*_), see text for details. The results of fitting the simulated diffusion MRI data using a cumulant expansion expression are presented for centralized eigenvalues **(C)** and *FA* **(D)**. The black and red symbols correspond to points where the DTI model explains more (black) or less (red) than 95% of the signal variation. Human neocortical neurons from the NeuroMorpho.org database were used for this analysis. The eigenvalues are in units of μm^2^/ms.

Centralized eigenvalues of the diffusion tensor are plotted against the corresponding centralized eigenvalues of the scatter matrix for all human neocortex neurons from the NeuroMorpho.org database in Figure 5A. There is a high degree of correlation apparent in the plot, consistent with the predicted relationship between diffusion tensor and the orientation distribution matrix given in Equation (5). However, there is also a systematic deviation. Specifically, the small eigenvalues of the diffusion tensor tend to be larger than or equal to the prediction based on the Gaussian model, and the primary eigenvalues tend to be smaller than or equal to the prediction based on the Gaussian model. As a consequence of the “less extreme” eigenvalues of the simulated diffusion tensor, *FA*_*D*_ tends to be smaller than or equal to its predicted value of *D*_*A*_*FA*_*T*_(∑^3^_*i* = 1_τ^2^_*i*_/∑^3^_*i* = 1_λ^2^_*i*_)^1/2^.

This systematic deviation is likely due to the DTI model being too crude an approximation for the diffusion MR signal to model the computed MR signal at the applied diffusion weighting. Specifically, the expression used to compute diffusion tensor eigenvalues in Figures 5A,B involve the assumption that the water displacement propagator is a Gaussian function of position. Under conditions of restricted diffusion, for example, this assumption is known to be only approximately true, with deviations from Gaussian behavior being larger with increasing diffusion weighting (Jensen et al., 2005; Kiselev, 2011). To demonstrate the link between inaccuracy of the Gaussian approximation and the systematic deviations observed in Figure 5, the quality of fit of the DTI model is indicated by the color of the Figure 5 data points. Black data points in Figures 5A,B are those for which the fitted diffusion MR signal explains more than 95% of the variation in the simulated diffusion signal, i.e. as computed by the diffusion model in Equation (2). In contrast, the data points in which the explanation degree is less than 95% have been plotted in red. The poor-fitting red data points deviate further from the line of unit slope than do data points that are more accurately approximated by Equation 2.

**Figure 6:**
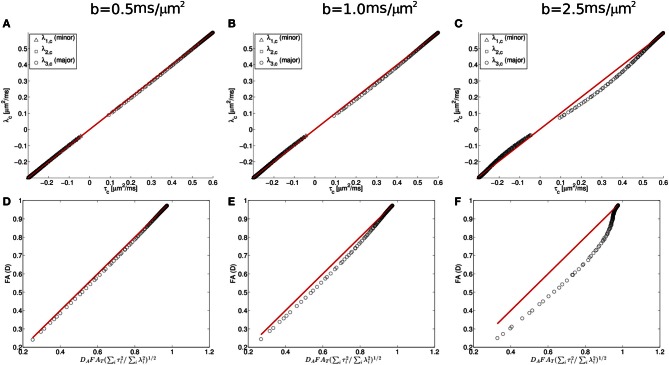
**Diffusion and scatter matrix centralized eigenvalues (λ and τ, respectively) plotted for simulated data (**A–C**; see text for details) and fractional anisotropy computed from the centralized eigenvalues for the diffusion tensor and orientation distribution matrices **(D–F)**.** These data demonstrate that deviations in the predicted relationship between the diffusion tensor matrix (D) and the orientation distribution matrix (T) are found when the strength of the diffusion-sensitizing magnetic field gradients is increased.

To further characterize this deviation, we consider next the simulation results from the Watson distribution of long and narrow cylinders. Consistent with the pattern shown in Figure 5, the simulation results determined from the Watson distribution (Figure 6) clearly display similar systematic deviations of the eigenvalues from the Gaussian model. As shown in Figure 5, the centralized eigenvalues of the diffusion tensor are “less extreme” than the scatter matrix eigenvalues, resulting in smaller *FA*_*D*_ values than *D*_*A*_*FA*_*T*_(∑^3^_*i* = 1_τ^2^_*i*_/∑^3^_*i* = 1_λ^2^_*i*_)^1/2^ shown in Figures 5A,D. Moreover, these systematic trends become more pronounced when the effects of restricted diffusion are accentuated by increasing the strength of the diffusion-sensitizing magnetic field gradients. For *b* = 0.5 ms/μm^2^ the simulated eigenvalues nearly coincide with the Equation (1.5) prediction (shown as a line in Figure 5), with the maximal difference between the predicted and observed *FA*_*D*_ being 0.0173 at an *FA*_*D*_ value of 0.522. Increasing deviations are found when increasing the *b*-value to 1 and 2.5 ms/μm^2^ (Figures 6B,E and 6C,F, respectively), in which maximal differences between observed and predicted *FA*_*D*_ values being 0.040 at *FA*_*D*_ value 0.586, and 0.142 at *FA*_*D*_ value 0.580, respectively.

The Gaussian approximation can be viewed as the first term in the cumulant expansion, a systematic series expansion in diffusion weighting *b* of the log diffusion signal (Kiselev, 2011). Retaining the next term in the cumulant expansion corresponds to the so-called diffusion kurtosis imaging, which is a method that has been used previously to account for the effects of non-Gaussian displacements in diffusion MRI (Liu et al., 2004; Jensen et al., 2005; Lu et al., 2006; Jensen and Helpern, 2010; Kiselev, 2011), resulting in a more accurate description of the diffusion signal over a wider range of diffusion weightings. The additional degrees of freedom in this expression relative to a diffusion tensor have also been found to yield more accurate estimates of the diffusion tensor (Veraart et al., 2011). The results of fitting the simulated diffusion MRI data using a second order cumulant expansion expression [Equation (1.4)] are presented for centralized eigenvalues and *FA* in Figures 5C,D, respectively. As expected, improved agreement with the Equation (1.5) prediction is observed in Figures 5C,D than in the corresponding Figure 5 panels **A** and **B**, as made evident by the narrower confidence bands (dashed lines in Figure 5), because the cumulant expansion model provides improved fitting of diffusion MRI data affected by restrictive barriers than does the DTI model.

## Discussion

In this work, we have utilized the NeuroMorpho database to characterize the expected diffusion MRI signal derived from water within geometries representative of real neocortical neurons. This comparison was achieved by combining the structural information in the database with a simple analytical model of diffusion in cylinders. The fact that the reconstruction data enables a complete characterization of single neuron structure provides a unique situation, in which fundamental questions about the microstructural underpinnings of diffusion MRI can be addressed in a realistic setting where ground truth is known. In the present paper, we have focused on the relationship between anisotropy in the orientation distribution matrix and anisotropy in water diffusivity in neocortical neurons. We focus on neurons in this GM structure because diffusion anisotropy measurements in neocortex have specifically been related to anisotropy in neuron morphology, organized around a primary apical dendrite. In principle, however, this approach could be extended to any GM structure in which water diffusion anisotropy is observed. We find that the dependence of neuron process orientation distribution anisotropy on the line length used to parameterize neurons is weak (0.002 μm^2^/ms per μm) over the range from 5 to 25 μm. Thus, it was concluded to be acceptable to continue previous practices (see Jespersen et al., 2012) of utilizing a 10 μm line length in approximations of neural structures.

From the standpoint of comparing neuron morphology between different neuron types and species, it was interesting to find considerable variability in scatter matrix anisotropy within the reconstructions available through the NeuroMorpho database. Significant differences in *FA*_*T*_ were revealed through ANOVA for different neuron types, with bipolar, and double bouquet neurons exhibiting highest anisotropy, and stellate neurons showing the least amount of anisotropy. Significant inter-species differences in pyramidal neuron *FA*_*T*_, with cat neurons having lowest anisotropy, and monkey being characterized by the highest mean *FA*_*T*_, were also observed. This pattern does not parallel the phylogenic complexity of species, however. For example, the mean *FA*_*T*_ is lower for human than for monkey, and the mean *FA*_*T*_ for mouse and rat is higher than that of cat. There is a possibility that technical differences associated with standard tissue preparation procedures, for example, could be a (potentially dominant) contributor to these inter-species differences. The results of ANOVA for rat pyramidal neurons revealed significant inter-laboratory differences, which suggests that differences in experimental procedures adopted by different research groups can give rise to variability in the characteristics of reconstructed neuron structures. In principle, a multiple-factor ANOVA could add clarity to those factors that are most influential in neuron structure within the NeuroMorpho database. Unfortunately however, we were unable to conduct reliable multiple-factor analyses, because there was little overlap between laboratories in the species and neuron types studied, and this precluded our ability to quantify interactions between the proposed factors influencing *FA*_*T*_.

The results of the MR diffusion simulations were used to compare the orientation distribution estimated based on Equation 7 to that determined directly from neuronal reconstructions. For human neocortical neurons, a systematic deviation was observed between the MR-predicted and actual orientation distributions, such that the anisotropy in orientation distributions are erroneously predicted to be low (Figure 5B). This effect is due to both an underestimation of the primary eigenvalue, and overestimation of minor eigenvalues of the orientation matrices, and the systematic discrepancy is larger for intermediate *FA* values than for extreme *FA* (Figures 5A,B).

One factor that contributes to the difference between predicted and observed orientation matrix eigenvectors is the approximation that the MR signal decays as a mono-exponential function of b (the Gaussian approximation). However, the accuracy of the Gaussian approximation is influenced by the amount of diffusion weighting as well as the form of the neurite orientation distribution. Therefore, an additional series of calculations were performed specifically for Watson-distributed sets of neurites for multiple diffusion weighting conditions. The more pronounced discrepancy observed at higher *b*-values (Figure 6) supports that the Gaussian approximation is the source of the observed systematic deviations, because the effects of restricted diffusion, which lead to non-monoexponential decay in MR signal intensity with *b*-value, are larger at higher *b*-values. Previous work (Veraart et al., 2011), has demonstrated that more general alternatives to the DTI model of water diffusion, such as the cumulant expansion/kurtosis models (Liu et al., 2004; Jensen et al., 2005; Lu et al., 2006; Jensen and Helpern, 2010; Kiselev, 2011), facilitate improved accuracy in the description of water diffusion within tissue. Here we found that accounting for non-gaussian effects by incorporating higher-order cumulant expansion terms into the expression for water diffusion provided improved agreement between observed and MR-predicted neurite orientation distribution eigenvalues (Figures 5C,D).

Some limitations in our ability to use results obtained here in the interpretation of diffusion MR data obtained from biological tissue merit recognition. First, the NeuroMorpho database does not provide information related to the structure of the extracellular space. In ours and others previous work (Assaf et al., 2004; Jespersen et al., 2007; Alexander et al., 2010), a parameter representing the volume fraction of the compartment exhibiting local cylindrical symmetry has been made explicit. This has been equated to the volume fraction of the neuropil in our applications of diffusion MR to studies of brain GM (Jespersen et al., 2010, 2012). Herein, the volume fraction of the cylindrical compartment has been fixed at a value of 1, reflecting our exclusive focus on diffusion within neurites from individual neurons, rather than on tissue volume elements as in our previous work. Further, another assumption of our previously-described model concerns the slow exchange of water across neuronal cell membranes. The validity of this assumption is supported by the highly selective expression of aquaporins (membrane water channels) in astrocytes, but not in neurons (Amiry-Moghaddam and Ottersen, 2003). It is also consistent with MR (Quirk et al., 2003; He et al., 2012) and PET (Larson et al., 1987) studies indicating a neuronal residence time of several seconds compared to a typical diffusion time of tens of milliseconds in diffusion MR experiments. However, it was not possible to specifically characterize the effect of water exchange in the context of neuron reconstructions provided by the NeuroMorpho database. Last, the effect of myelin on water diffusion anisotropy, which has been proposed to influence water diffusion anisotropy even within GM structures such as the mature cerebral cortex (Leuze et al., 2012; McNab et al., 2013) could not be addressed in this study due to the lack of glial cells in the NeuroMorpho data. Thus, although the analyses presented here do provide a unique opportunity to characterize the influence of diffusion in known neuron structures on diffusion-weighted MR data, there are factors that influence water diffusion in tissue that could not be addressed in this study.

In conclusion, reconstructed neurons from the NeuroMorpho database were shown to span a wide range of scatter matrix anisotropy, making them suitable for extensive testing and model validation. Here we used them to verify a close relationship between the scatter matrix of neuronal structures and the diffusion tensors characterizing diffusion MRI, especially if care is taken to account for violations of Gaussian diffusion which affect the estimation of the diffusion tensor. These results will be helpful for a quantitative interpretation of GM diffusion anisotropy in terms of neuronal morphology.

### Conflict of interest statement

The authors declare that the research was conducted in the absence of any commercial or financial relationships that could be construed as a potential conflict of interest.

## Footnotes

^1^Reconstructions were downloaded from the following archives of NeuroMorpho.Org: “Allman,” “Barbour,” “Bergstrom,” “Bikson,” “Brown,” “Brumberg,” “Cauli,” “DeFelipe,” “Dendritica,” “Destexhe,” “Eysel,” “Gonzalez-Burgos,” “Helmstaedter,” “Hirsch,” “Hirsch, DIADEM,” “Jacobs,” “Kawaguchi,” “Kilb,” “Korngreen,” “Kubota,” “Lewis,” “Luebke,” “Markram,” “Martin,” “Meyer,” “Monyer,” “Nolan,” “Poorthuis,” “Povysheva,” “Smith,” “Staiger,” “Sun_Prince,” “Svoboda,” “Timofeev,” “Vuksic,” “Wearne_Hof,” and “Yuste”. A total of 62 publications are associated with these 37 Archives and could not be cited here, but are included in Appendix B

## Appendix A

### Estimating the transverse diffusion coefficient

In this appendix, we present the key points in deriving an expression for the transverse diffusion coefficient *D*_*T*_. Following the work of Stepišnik (1993), one may compute the diffusion of a single proton based on a cumulant expansion of the self-diffusion expression. We choose to truncate the expansion at second order, yielding

(A1) $$S(q,\;\Delta )=S_{0}e^{-bD_{T}}=S_{0}e^{-\beta (\Delta )}$$

where β (Δ) is the second order term in the cumulant expansion, and it has been used that the longitudinal part of the diffusion signal is zero, as we focus solely on diffusion orthogonal to the cylinder direction. In Equation (A1), the first equality is the signal corresponding to Equation (2) for a single proton diffusing transversely in a cylinder. The second equality models the phase of a single proton, and depends on the type of experiment performed, the boundaries if the diffusion compartment, etc., see Stepišnik (1993). Relating the left and right hand sides of the second equality sign, one obtains an expression for *D*_*T*_

(A2) $$D_{T}=\frac{1}{b}\beta (\Delta )$$

Following the derivations in Stepišnik (1993), we now express β (Δ) in terms of the longitudinal diffusivity *D*_*L*_ and the neurite radius *R*, as

(A3) $$\beta \left(\Delta \right)=2\left(\frac{\gamma G}{D_{L}}\right)\sum_{k}\frac{B_{k}}{a_{k}^{2}}\left[a_{k}D_{L}\delta -1+e^{-a_{k}D_{L}\delta }+e^{-a_{k}D_{L}\Delta }\left(1-\cosh\left(a_{k}D_{L}\delta \right)\right)\right]$$

where γ is the gyromagnetic ratio of the nuclei under consideration, *g* is the magnetic field gradient strength. In Equation (A3), *B*_*k*_ and *a*_*k*_ depend on the boundaries of the self-diffusion compartment. In the case of a cylindrical compartment of radius *R*, which is the relevant compartment in our case, one obtains (Stepišnik, 1993)

(A4) $$B_{k}=2\frac{{\left(R/\mu_{k}\right)}^{2}}{\mu_{k}^{2}-1}\text{ and }a_{k}={\left(\frac{\mu_{k}}{R}\right)}^{2}$$

where μ_*k*_ is the *k*'th root of the first derivative of the first order Bessel function of the first kind. It is now a simple task to combine Equations (A2), (A3), and (A4) to obtain an expression for the transverse diffusion coefficient in terms of a fixed radius and pulse parameters Δ and δ. The final expression being

(A5) $$D_{T}=4R^{6}{\left({\left(\delta D\right)}^{2}\left(\Delta -\delta /3\right)\right)}^{-1}\sum_{k}{\left(\mu_{k}^{6}\left(\mu_{k}^{2}-1\right)\right)}^{-1}[\frac{\mu_{k}^{2}D\delta }{R^{2}}-1+e^{-\frac{\mu_{k}^{2}D\delta }{R^{2}}}+e^{-\frac{\mu_{k}^{2}D\Delta }{R^{2}}}-\;\frac{1}{2}e^{-\frac{\mu_{k}^{2}D(\Delta \;-\;\delta )}{R^{2}}}+\frac{1}{2}e^{-\frac{\mu_{k}^{2}D(\Delta \;+\;\delta )}{R^{2}}}]$$

In which it has been used that *b* = (γ*g*δ)^2^ (Δ − δ/3). In this work the summation in Equation (A5) is truncated after the tenth root, which is deemed sufficient in terms of convergence of the series.
